# How face-like objects and averted gaze faces orient our attention: The role of global configuration and local features

**DOI:** 10.1177/20416695251352129

**Published:** 2025-07-23

**Authors:** Ziwei Chen, Mengxin Wen, Xun Liu, Di Fu

**Affiliations:** 1 Institute of Psychology, Chinese Academy of Sciences, Beijing, China; Department of Psychology, University of Chinese Academy of Sciences, Beijing, China; 2 School of Psychology, University of Surrey, Guildford, Surrey, UK; Surrey Institute for People-Centred AI, University of Surrey, Guildford, Surrey, UK

**Keywords:** face pareidolia, attentional shifts, averted gaze, global process, local process

## Abstract

In real life, people perceive nonexistent faces from face-like objects, called face pareidolia. Face-like objects, similar to averted gazes, can direct the observer's attention. However, the similarities and differences in attentional shifts induced by these two types of stimuli remain underexplored. Through a gaze cueing task, this study compares the cueing effects of face-like objects and averted gaze faces, revealing both commonalities and distinct underlying mechanisms. Our findings demonstrate that while both types of stimuli can elicit attentional shifts, the mechanisms differ: averted gaze faces rely on processing local features like gaze direction, whereas face-like objects leverage their global configuration to enhance attentional shifts by triggered eye-like features. These findings advance the understanding of the processing mechanisms underlying the perception of face-like objects, and how the brain represents facial attributes even when physical facial stimuli are absent. This study provides a valuable theoretical foundation for future investigations into the broader applications of face-like stimuli in human perception and attention.

## How to cite this article

Chen Z., Wen M., Liu X., & Fu D. (2025). How face-like objects and averted gaze faces orient our attention: The role of global configuration and local features. *i-Perception*, *16*(4), 1–20. https://doi.org/10.1177/20416695251352129

## Introduction

Individuals often perceive nonexistent faces from meaningless and ambiguous objects, a phenomenon known as face pareidolia (Palmer & Clifford, 2020; Takahashi & Watanabe, 2013). The detection of face pareidolia involves both local and global processes, engaging both object perception and higher-order cognitive functions (Akdeniz, 2023; Leadner et al., 2022; Lhotka et al., 2023; Wardle et al., 2023). Perceiving face-like objects is associated with increased prosocial behavior (Hamza et al., 2022), influencing individual attention allocation and diversion (Castello et al., 2014; Henschel et al., 2021; Jakobsen et al., 2023; Keys et al., 2021). For instance, architecture and paintings incorporating face pareidolia can capture attention (Martinez-Conde et al., 2015; Wang et al., 2022). Additionally, products incorporating face pareidolia can guide consumers’ attention to increase their purchasing behaviors (Delbaere et al., 2011; DiSalvo & Gemperle, 2003; Guido et al., 2018; Noble et al., 2023; Wodehouse et al., 2018). However, the mechanisms by which face-like objects trigger attentional shifts remain unclear, as does the distinction between these shifts and those induced by common averted gaze faces.

The low-level physical properties and high-level social attributions of face-like objects likely contribute to attentional shifts. From a physical perspective, individuals might perceive the orientation of objects based on large-scale variations in luminance or asymmetry. However, few studies have investigated the impact of the objects’ orientation on triggering attentional shifts. From a social perspective, individuals might attribute social meanings to these objects, allocating attention similarly to how they would respond to averted gazes or head orientation. Averted gazes often prompt individuals to look in the direction of the gaze to obtain social information (Frischen et al., 2007; Ishikawa et al., 2021; Sweeny & Whitney, 2014; Tanaka et al., 2022). Given that the shape and the contrast between the inner and outer parts of these eye-like parts of objects resemble those of real eyes (Burra & Kerzel, 2021; Epley et al., 2007), individuals could associate the eye-like features of objects with averted gaze, orienting their attention accordingly (Kobayashi & Kohshima, 2001; Zhang et al., 2011). Head orientation has been shown not only to trigger attentional shifts (Hoehl et al., 2014; Tautvydaite & Burra, 2024) but also to amplify the effect of an averted gaze (Jenkins & Langton, 2003; Kluttz et al., 2009; Qian et al., 2013; Schwaninger et al., 2005; Todorovic, 2006). When individuals see face-like objects, they are likely to mentally imbue these face-like configurations with social attributes, particularly those related to head orientation. This tendency raises an intriguing question: Do the attentional shifts triggered by face-like objects stem primarily from their physical properties or from the social attributions that individuals unconsciously imbue to them? Our investigation explores this dichotomy, examining whether it is the eye-like parts or the face-like configuration that primarily drives these attentional shifts.

This study employed a gaze cueing task to investigate these questions. Unlike the Posner cueing task, which uses directional arrows (Posner, 1980), the gaze cueing task is a well-established paradigm for investigating the attentional underpinnings of attentional shifts triggered by averted gazes (Edwards et al., 2015; Friesen & Kingstone, 1998). Typically, an averted gaze face is presented as the cueing stimulus at the center of the screen. Previous studies have used various types of averted gaze faces, including real faces (Ji et al., 2020; Le et al., 2021; Lin et al., 2020; Liu et al., 2021; McCrackin & Itier, 2018; van Rooijen et al., 2018; Vivanti et al., 2017), computer-generated faces (Caruana et al., 2019; Ji et al., 2022), and schematic faces (Birmingham & Kingstone, 2009; Dodd et al., 2011; Friesen & Kingstone, 1998; Wei et al., 2019; Yokoyama et al., 2019; Yokoyama & Takeda, 2019). After a short stimulus onset asynchrony of 200 to 350 ms (Conty et al., 2006; McCrackin & Itier, 2018; Yoxon et al., 2019), a geometric pattern or a light spot is presented as the target stimulus on either the left or right side of the screen (Ishikawa et al., 2021; Ji et al., 2020). Participants usually need to respond to the location of the target stimuli (Friesen & Kingstone, 1998; Frischen et al., 2007; Ishikawa et al., 2021; Ji et al., 2020, 2022), they can also perform detection or identification tasks (Bayliss et al., 2011; Burra & Kerzel, 2021; Friesen & Kingstone, 1998). The use of a localization task is often chosen because cueing effects tend to be larger in such tasks (McKay et al., 2021). Even when participants are explicitly informed that the direction of the cueing stimuli does not predict the location of the subsequent target stimuli, they still respond faster in congruent trials (where the target was presented at the pointing-to locations) than in incongruent trials (where the target was not presented at the pointing-to locations; Friesen & Kingstone, 1998). The gaze cueing effect refers to the difference in response time (RT) between congruent and incongruent trials (calculated as the mean incongruent RTs minus the mean congruent RTs; Ishikawa et al., 2021; Talipski et al., 2020). The gaze cueing effect is influenced by several factors. For instance, the cueing effect disappears when the duration of the cueing stimuli exceeds 1,000 ms, a phenomenon explained by the inhibition of return (Frischen et al., 2007; Yoxon et al., 2019). Additionally, some studies have found a positive correlation between the extent of gaze direction and the strength of the gaze cueing effect (Yokoyama & Takeda, 2019; Zhang et al., 2011). Consequently, researchers often control for perceived gaze direction when using the gaze cueing task (Bayliss et al., 2011; Qian et al., 2013).

In a gaze cueing task, the gaze cueing effect is a crucial measure of attentional shifts triggered by averted gazes. This effect, defined as the difference in RT between congruent and incongruent trials, is attributed to attentional shifts (Atkinson et al., 2018; Ji et al., 2022; Talipski et al., 2020). Notably, some studies have found no difference in cueing effects elicited by averted gaze faces and directional arrows (Bayliss et al., 2011; Lin et al., 2020; Tipples, 2002). However, other research has shown that averted gaze faces generate a stronger cueing effect than arrows, with individuals associating this asymmetry with directional information (Gregory & Jackson, 2017; Ristic et al., 2012). This suggests the involvement of a social-special mechanism based on social characteristics rather than a domain-general attention mechanism (Bayliss et al., 2011; Dalmaso et al., 2020). The enhanced cueing effect may stem from the heightened saliency, ecologically valid signals, or social-biological information in averted gaze faces compared to directional arrows (Birmingham & Kingstone, 2009; Ishikawa et al., 2021; Tipper & Bayliss, 2018). Besides, dividing attentional shifts into attentional benefit and cost can be challenging when cue-probe congruency is limited to two conditions (congruent vs. incongruent; Bayliss et al., 2011; Yokoyama & Takeda, 2019). Friesen and Kingstone (1998) used schematic faces as cueing stimuli and introduced directed gaze faces as neutral trials. They found that cue-probe congruency produced an attentional benefit without a cost and suggested that the schematic faces used as the cueing stimuli might weaken the gaze cueing effect. Subsequent research often employs real faces (Ji et al., 2020; Le et al., 2021; Lin et al., 2020; Liu et al., 2021; McCrackin & Itier, 2018; van Rooijen et al., 2018; Vivanti et al., 2017) or computer-generated faces (Caruana et al., 2019; Ji et al., 2022) as cueing stimuli. The gaze cueing effect can be more comprehensively elucidated by using these faces and comparing RTs across all three conditions of cue-probe congruency.

Our study explores the similarities and differences in attentional shifts induced by face-like objects and averted gaze faces. Investigating attentional shifts induced by face-like objects can reveal the underlying processing mechanisms of these objects, specifically what information individuals extract to make these shifts (Atkinson et al., 2018; Palmer & Clifford, 2020; Rahman & Boxtel, 2022). Recent studies have explored the role of face-like objects in attentional diversion. For instance, Takahashi and Watanabe (2013) used face-like objects and schematic faces as cueing stimuli and found no differences in cueing effects between them. Two potential explanations might account for their findings: First, the schematic faces lacked ecological validity compared to real faces (Friesen & Kingstone, 1998; Le et al., 2021; Liu et al., 2021; van Rooijen et al., 2018) or computer-generated faces (Caruana et al., 2019), potentially resulting in weaker cueing effects. Second, the study did not control gaze direction, which influenced the magnitudes of the gaze cueing effects for both face-like objects and schematic faces. Our empirical investigations for verifying these explanations proceed as follows. In a preliminary experiment, we measured the directions of averted gaze faces and face-like objects, categorizing them as two different cueing stimuli for subsequent experiments (see Supplemental Information). In Experiment 1, we used the gaze cueing task to test whether cueing effects in face-like objects would be similar to those observed in averted gaze faces. Nonetheless, the role of low-level physical properties, eye-like features, or face-like configurations in triggering attentional shifts remained undetermined. Thus, in Experiment 2, we isolated the averted gazes and eye-like parts of the two stimuli, and in Experiment 3, we presented the two stimuli in an inverted form. Finally, we compared cueing effects among three presenting modes in Experiment 4 to investigate these issues.

## Experiment 1

Based on previous studies (Friesen & Kingstone, 1998), we introduced a category of neutral cueing stimuli to expand cue-probe congruency. This approach allowed us to classify the cueing effect as either an attentional benefit (RTs in congruent trials < RTs in neutral trials) or an attentional cost (RTs in neutral trials < RTs in incongruent trials). We proposed two hypotheses for this experiment. First, if cueing stimuli induce heightened arousal levels in participants, we hypothesized they would exhibit faster reaction times (RTs) in both congruent and incongruent trials compared to neutral trials (Aston-Jones et al., 1999; Ishikawa et al., 2021). Second, if participants allocate attentional resources specifically to the cued location, we hypothesized distinct patterns based on the underlying mechanism: For attentional facilitation, responses would be fastest in congruent trials, followed by neutral trials, and slowest in incongruent trials; For attentional inhibition, responses would be fastest in neutral trials, followed by congruent trials, and slowest in incongruent trials (Fu et al., 2023). Critically, we predicted that these attentional allocation patterns would apply to both real faces and face-like objects (following Takahashi & Watanabe, 2013).

### Material and Methods

#### Participants

A priori power analysis using G*Power (Version 3.1.9.7) indicated that a sample size of 17 participants would be required to achieve a power of 95% for detecting a medium effect size in a two-factor (2 × 3) repeated measures ANOVA (*f* = 0.33) at a significance criterion of *α* = 0.05. When considering the interaction of multiple within-subject variables, the estimation of degrees of freedom from the off-center distribution becomes inaccurate, leading to a substantial underestimation of the required sample size. Since G*Power is more suitable for single within-subject designs, we recalculated the sample size using MorePower (Campbell & Thompson, 2012), which suggested that 46 participants would be needed. Fifty-four undergraduate and graduate students (28 self-identified as female, 26 as male, 0 as nonbinary; *M* ± *SD*_age_ = 21.28 ± 2.56 years; range 18–27) participated in Experiment 1. All participants were right-handed, of Asian ethnicity, reported having normal color vision, and provided informed consent.

#### Apparatus and Stimuli

Experiment 1 was performed using E-prime 2.0 (Psychology Software Tools, Inc., Sharpsburg, PA, USA) for programming and data recording. Stimuli were presented on a 21.5-inch Dell display (1024 × 768 at 60 Hz), 32-bit color depth.

**Figure 1.**
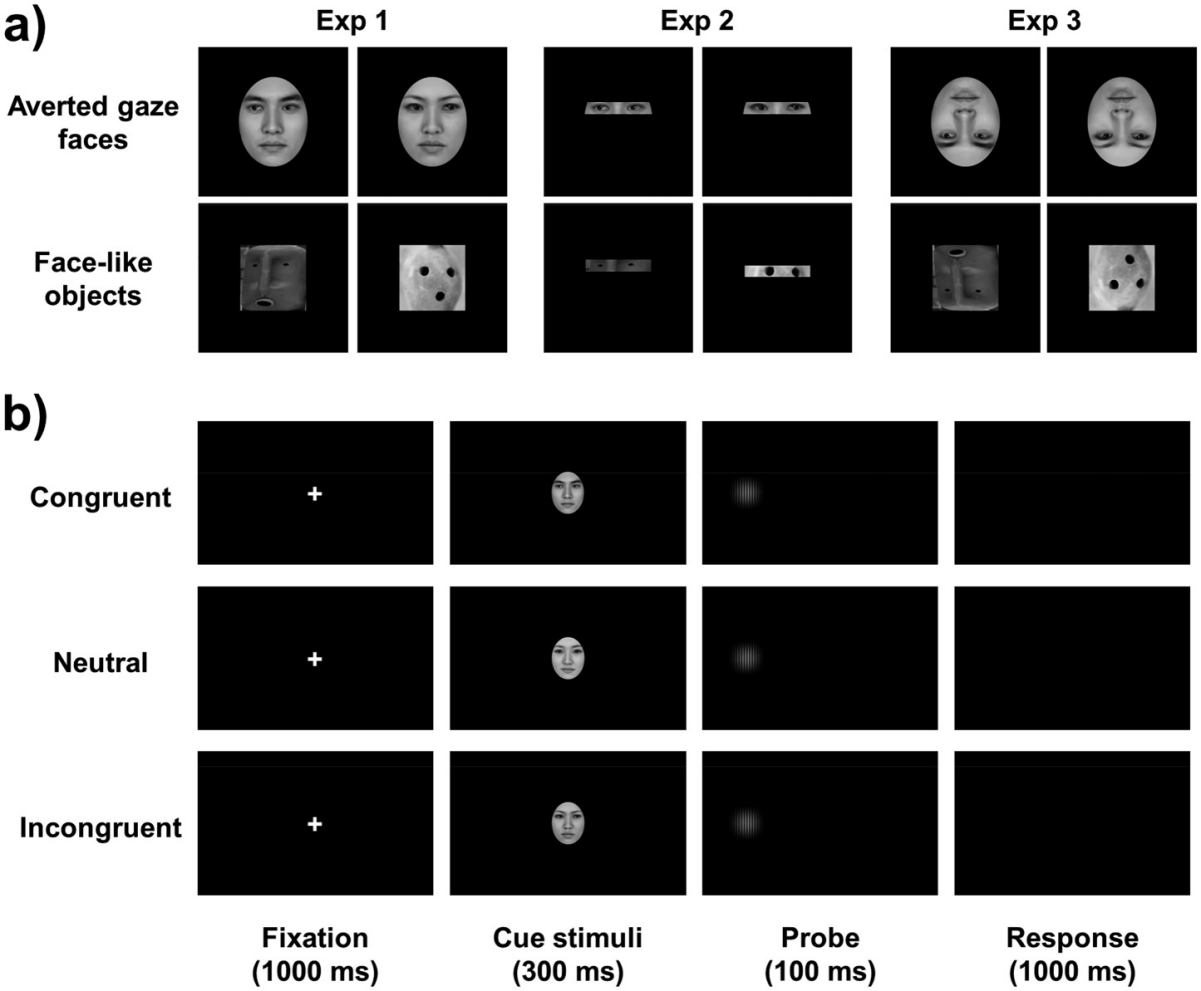
Flow diagram for Experiments 1 to 3. (a) The cueing stimuli used in these experiments, including averted gave faces and face-like objects. (b) The procedure for a single trial when using averted gaze faces as cueing stimuli.

Based on the preliminary experiment, we employed three directions of cueing stimuli: averted gaze faces and face-like objects looking directly at the viewer, and looking to the right or left side (see Supplemental Figures S1–S3). Each direction of averted gaze face stimuli consisted of 12 faces (half male faces and half female faces). Each direction of face-like object stimuli consisted of 12 objects. Examples of the cueing stimuli are shown in Figure 1a. The stimuli used in this study are publicly available on the Open Science Framework at https://osf.io/h79ns/.

#### Procedure

Based on previous studies, the procedure for one trial is shown in Figure 1b. Each trial started with a fixation “+” (1.1° × 1.1°), lasting for 1,000 ms in the center of the screen. Then, a cueing stimulus (9.1° × 9.1°) was displayed for 300 ms in the center of the screen. After a cueing stimulus disappeared, a Gabor patch (4.3° × 4.3°) was presented as the target stimulus for 100 ms, with a 50% probability on either the left or right side (16.7° from the center of the screen). Following the target stimulus, a black screen was displayed for 1,000 ms. Participants were instructed to press one of two keys as quickly and accurately as possible to indicate the location of the target stimulus (F-key for the left and J-key for the right). At the beginning of the experiment, participants were explicitly informed that the cueing stimuli did not predict the target location. There were two blocks, each containing one type of cueing stimuli. Each block consisted of 360 trials: 120 congruent trials (the direction of cueing stimuli was the same as the location of target stimuli), 120 incongruent trials (the direction of cueing stimuli was opposite as the location of target stimuli), and 120 neutral trials (the direction of cueing stimuli was straight to the viewer). The number of target-left and target-right trials was equal across congruent, neutral, and incongruent trials. The order of the two blocks was counterbalanced across participants (nine women and six men did the face block first). The entire experiment took about 20 min.

#### Data Analysis

The mean accuracy (ACC) was 95.81%. Therefore, we did not compare accuracy across conditions. For RT analysis, we excluded trials with RTs more than 3 SDs above or below the mean, resulting in an average exclusion of 8.59 trials per participant. The remaining mean RTs were subjected to a two-factor repeated-measures ANOVA with stimuli type (averted gaze faces vs. face-like objects) and cue-probe congruency (congruent vs. neutral vs. incongruent). Bonferroni corrections were adopted for all comparisons. The data in the current study are publicly available on the Open Science Framework at https://osf.io/h79ns/.

### Results

**Figure 2.**
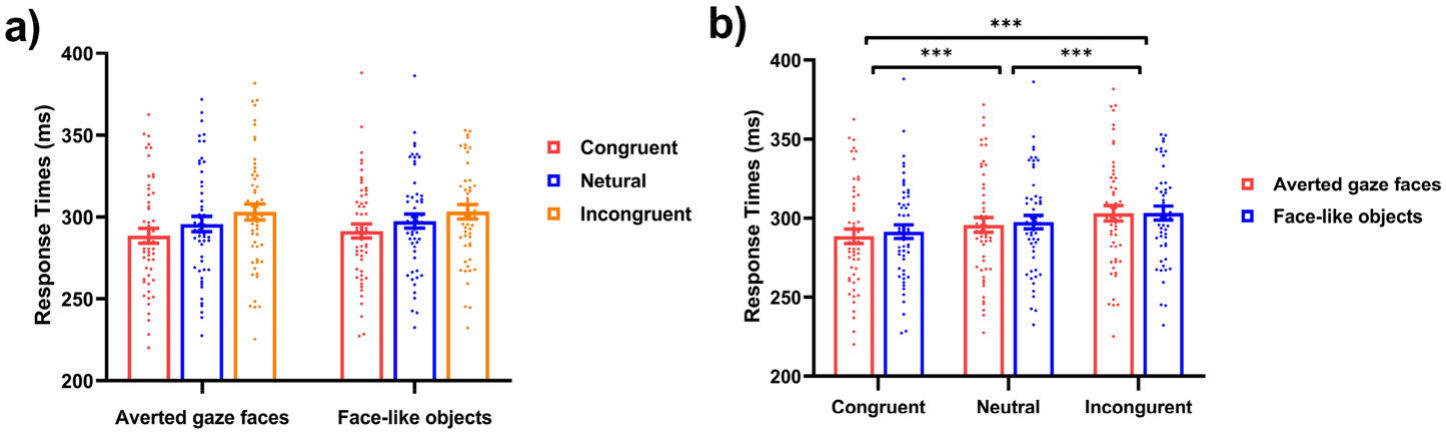
Results of mean RTs in Experiment 1. (a) Mean RTs for the gaze cueing task as a function of cue-probe congruency (congruent vs. neutral vs. incongruent). (b) Mean RTs for the gaze cueing task as a function of stimulus type (averted gaze faces vs. face-like objects).Error bars represent standard errors. *** *p* < .001, ** *p* < .01, * *p* < .05.

*Note.* RT = response time.

For RTs, the ANOVA analysis revealed a nonsignificant main effect of stimuli type (averted gaze faces vs. face-like objects), *F*(1, 53) = 0.721, *p* = .400, $\eta_{p}^{2}$ = .013. This indicates that there was no statistically significant difference in RTs between the averted gaze faces (295.75 ± 33.94 ms) and face-like objects (297.34 ± 31.27 ms). And a significant main effect of cue-probe congruency (congruent vs. neutral vs. incongruent) was observed, *F*(2, 106) = 106.094, *p* < .001, $\eta_{p}^{2}$ = .667. Post hoc testing further elucidated that compared with incongruent trials (303.06 ± 33.57 ms), participants responded significantly faster both in congruent trials (290.01 ± 32.23 ms, *p* < .001) and in neutral trials (296.56 ± 32.73 ms, *p* < .001), and participants responded significantly faster in congruent trials than in neutral trials (*p* < .001). There was no significant interaction between stimuli type and cue-probe congruency, *F*(2, 106) = 1.999, *p* = .141, $\eta_{p}^{2}$ = .036. The mean RTs from six conditions are shown in Figure 2.

### Discussion

Overall, the results in Experiment 1 suggested that both averted gaze faces and face-like objects can trigger attentional shifts. Participants responded more quickly in congruent trials compared to incongruent trials. The inclusion of neutral cueing stimuli revealed significant differences in RTs among the three conditions, further confirming that the main effect of cue-probe congruency resulted from attentional shifts (Atkinson et al., 2018; Ji et al., 2022; Talipski et al., 2020).

We also observed a cueing effect in face-like objects, consistent with previous studies (Takahashi & Watanabe, 2013). Studies suggested that the primary visual signals of face-like objects were often incomplete and ambiguous (Taubert et al., 2017; Wang et al., 2022). Consequently, the brain automatically uses prior experiences and knowledge to fill in the missing components, constructing a coherent and comprehensive image. When processing face-like objects, the brain compares primary visual signals with suitable analogs and associates these signals with attributes stored in memory (Bar, 2007; Chen et al., 2023; Hamza et al., 2022; Rekow et al., 2022), influencing subsequent judgments. Individuals likely use the information of averted gazes stored in the brain to fill in the missing parts of these objects’ ambiguous visual signals, thereby perceiving gaze direction and making attentional shifts.

## Experiment 2

In Experiment 1, cueing effects were observed for both types of stimuli. Notably, the cueing effect persisted in averted gazes even when the global configuration was removed (Burra & Kerzel, 2021; Conty et al., 2006; Senju et al., 2005; Tanaka et al., 2022). This suggested that the interpretation of gaze direction primarily relies on local features within the eye region (Burra & Kerzel, 2021; Senju et al., 2008; Tanaka et al., 2022). Similarly, when processing face-like objects, individuals might perceive orientation based on the pronounced contrast within eye-like features (Burra & Kerzel, 2021; Epley et al., 2007). To this end, we selectively retained only the eyes or eye-like parts within the cueing stimuli. According to previous studies, we hypothesized cueing effects would persist in both averted gazes and eye-like features. If confirmed, this outcome would imply that individuals interpret gaze direction from these eye-like features, thereby facilitating attentional shifts. If no attentional benefit or cost is found in the eye-like features of face-like object stimuli, it may indicate that participants do not extract sufficient directional information from them.

### Material and Method

#### Participants

Forty-eight undergraduate and graduate students (29 self-identified as female, 19 as male, 0 as nonbinary; *M* ± *SD*_age_ = 23.85 ± 2.62 years; range 18–30 years) participated in Experiment 2. All participants were right-handed, of Asian ethnicity, reported having normal color vision, and provided informed consent.

#### Apparatus and Stimuli

The apparatus and stimuli used in Experiment 2 were the same as those in Experiment 1 except for the following changes. The cueing stimuli of averted gaze faces presented only the eye parts and did not contain eyebrows, and the cueing stimuli of face-like objects presented only the eye-like parts, as shown in Figure 1a.

#### Procedure

The procedure of Experiment 2 was identical to Experiment 1 (12 women and 11 men did the face block first).

#### Data Analysis

The mean accuracy (ACC) was 96.27%. Therefore, we did not compare accuracy across conditions. For RT analysis, we excluded trials with RTs more than 3 SDs above or below the mean, resulting in an average exclusion of 9.29 trials per participant. The remaining mean RTs were subjected to a two-factor repeated-measures ANOVA with stimuli type (averted gaze faces vs. face-like objects) and cue-probe congruency (congruent vs. neutral vs. incongruent). Bonferroni corrections were adopted for all comparisons.

### Results

**Figure 3.**
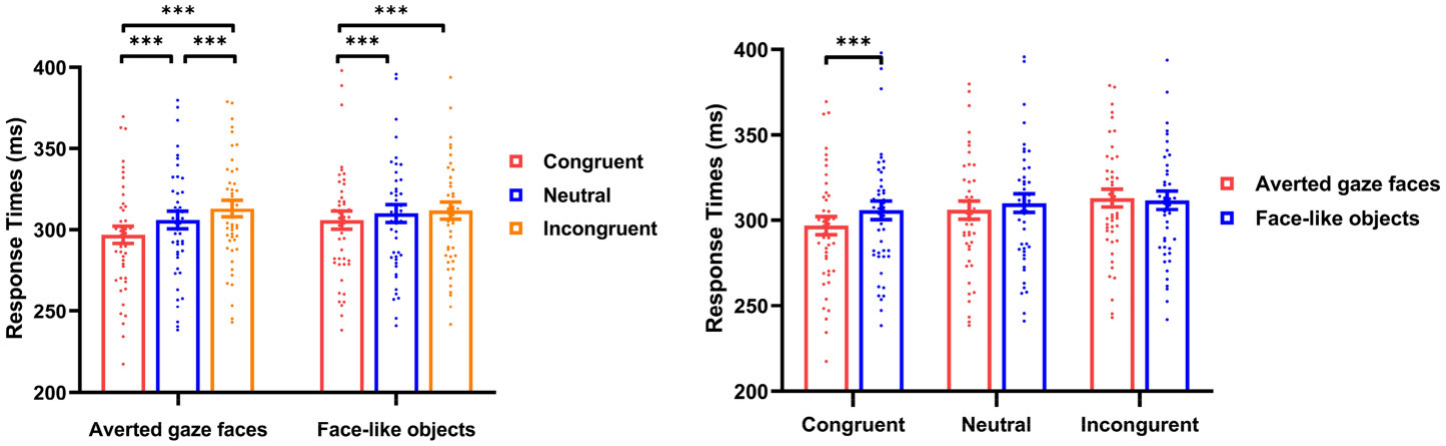
Results of mean RTs in Experiment 2. Mean RTs for the gaze cueing task as a function of stimuli type (averted gaze faces vs. face-like objects) and cue-probe congruency (congruent vs. neutral vs. incongruent). Error bars show standard errors. *** *p* < .001, ** *p* < .01, * *p* < .05.

*Note.* RT = response time.

For RTs, the ANOVA analysis revealed a nonsignificant main effect of stimuli type (averted gaze faces vs. face-like objects), *F*(1, 47) = 2.799, *p* = .101, $\eta_{p}^{2}$ = .056. This indicates that there was no statistically significant difference in RTs between the averted gaze faces (305.26 ± 36.55 ms) and face-like objects (309.19 ± 38.06 ms). And a significant main effect of cue-probe congruency (congruent vs. neutral vs. incongruent) was observed, *F*(2, 94) = 76.697, *p* < .001, $\eta_{p}^{2}$ = .620. Post hoc testing further elucidated that compared with incongruent trials (303.06 ± 35.91 ms), participants responded significantly faster both in congruent trials (290.01 ± 36.73 ms, *p* < .001) and in neutral trials (296.56 ± 37.13 ms, *p* < .001), and participants responded significantly faster in congruent trials than in neutral trials (*p* < .001). There was a significant interaction between stimuli type and cue-probe congruency, *F*(2, 94) = 30.248, *p* < .001, $\eta_{p}^{2}$ = .392. The mean RTs from six conditions are shown in Figure 3.

Considering the interaction between stimuli type and cue-probe congruency, a simple effect analysis revealed that for averted gaze faces, the RTs in congruent trials (269.77.55 ± 36.72 ms) were lower than in neutral trials (305.93 ± 37.59 ms; *t*(47) = –9.605, *p* < .001, *Cohen's d* = –1.386) and in incongruent trials (313.07 ± 36.14 ms; *t*(47) = −11.525, *p* < .001, *Cohen's d* = –1.664), with differences between the latter (*t*(47) = –7.886, *p* < .001, *Cohen's d* = –1.138). For face-like objects, the RTs in congruent trials (305.81 ± 38.67 ms) were lower than in neutral trials (310.00 ± 38.61 ms; *t*(47) = –4.596, *p* < .001, *Cohen's d* = –0.663) and in incongruent trials (311.77 ± 37.70 ms; *t*(47) = –4.576, *p* < .001, *Cohen's d* = –0.660). Furthermore, we also found the RTs for averted gaze faces were lower than for face-like objects in congruent trials (*t*(47) = –3.674, *p* < .001, *Cohen's d* = –0.530).

### Discussion

Cueing effects were observed for both averted gazes and eye-like parts stimuli, confirming that individuals interpreted gaze direction based on the local features within the eyes or eye-like parts. Nevertheless, the results indicated distinct main effects of cue-probe congruency between these two types of stimuli. Specifically, averted gaze stimuli produced both attentional benefit and cost, whereas eye-like parts stimuli resulted solely in attentional benefit. Previous research found that children fixated longer on upright rather than inverted face-like objects (Guillon et al., 2016), indicating that the global configuration can promote the representation of face-like objects (Chen et al., 2023; Leadner et al., 2022; Zhou et al., 2021). Therefore, participants probably perceived weaker directional information from eye-like parts due to the absence of global configuration.

Another potential explanation could be related to the viewing angles of these stimuli. Unlike averted gaze faces, which were frontally oriented toward the viewer, some face-like objects in this study may have varied in orientation due to differences in viewing angles. Additionally, other global configurations, such as larger-scale luminance variations, might also convey directional information. The absence of global configuration may account for the observed differences in the main effects of cue-probe congruency between the two stimulus types. To further investigate this hypothesis, we conducted Experiment 3.

## Experiment 3

Experiment 1 confirmed that face-like objects can trigger attentional shifts, consistent with previous findings (Takahashi & Watanabe, 2013). Experiment 2 revealed a cueing effect for eye-like parts, suggesting that individuals can derive directional information from these features. However, the specific role played by the global configuration of face-like objects in triggering attentional shifts was not identified. To address this question, we conducted Experiment 3, in which we inverted the cueing stimuli to disrupt the facial configuration (Bartlett & Searcy, 1993; Schwaninger et al., 2005). A main effect of cue-probe congruency in inverted face-like objects would suggest that attentional shifts induced by face-like objects depend on the facial configuration. Similar to the hypothesis of Experiment 2, if no attentional benefit or cost is found in inverted face-like object stimuli, it may indicate that participants do not extract sufficient directional information from them.

Furthermore, a cross-experiment comparison was conducted to investigate the differences in cueing effects induced by these two types of stimuli across the three experiments. If the cueing effects of upright face-like objects were greater than those of eye-like parts or inverted face-like objects, the results would suggest that the global process facilitated subsequent attentional shifts. Conversely, if the cueing effects of inverted face-like objects were greater than those of eye-like parts, it would suggest that individuals primarily perceive orientation based on the high contrast and large-scale luminance variations.

### Material and Method

#### Participants

Fifty-two undergraduate and graduate students (31 self-identified as female, 21 as male, 0 as nonbinary; *M* ± *SD*_age_ = 24.33 ± 1.51 years; range 21–28 years) participated in Experiment 3. All participants were right-handed, of Asian ethnicity, reported having normal color vision, and provided informed consent.

#### Apparatus and Stimuli

The apparatus and stimuli used in Experiment 3 were the same as in Experiment 1 except for the following changes. The cueing stimuli were inverted, as shown in Figure 1a.

#### Procedure

The procedure of Experiment 3 was identical to Experiment 1 (18 women and nine men did the face block first).

#### Data Analysis

The mean accuracy (ACC) was 96.81%. Therefore, we did not compare accuracy across conditions. For RT analysis, we excluded trials with RTs more than 3 SDs above or below the mean, resulting in an average exclusion of 8.87 trials per participant. The remaining mean RTs were subjected to a two-factor repeated-measures ANOVA with stimuli type (averted gaze faces vs. face-like objects) and cue-probe congruency (congruent vs. neutral vs. incongruent). Bonferroni corrections were adopted for all comparisons.

According to previous studies, the cueing effect referred to the difference in RTs between the congruent and incongruent trials (Ishikawa et al., 2021; Talipski et al., 2020). To investigate whether the cueing effects (mean incongruent RTs minus mean congruent RTs) found in two stimuli persisted in Experiment 1 (upright), Experiment 2 (eye-only) and Experiment 3 (inverted), we compared the cueing effects of different stimuli in three experiments. The cueing effects were subjected to a two-factors mixed ANOVA with stimuli type (averted gaze faces vs. face-like objects) and stimuli presenting mode (upright vs. eye-only vs. inverted).

### Results

**Figure 4.**
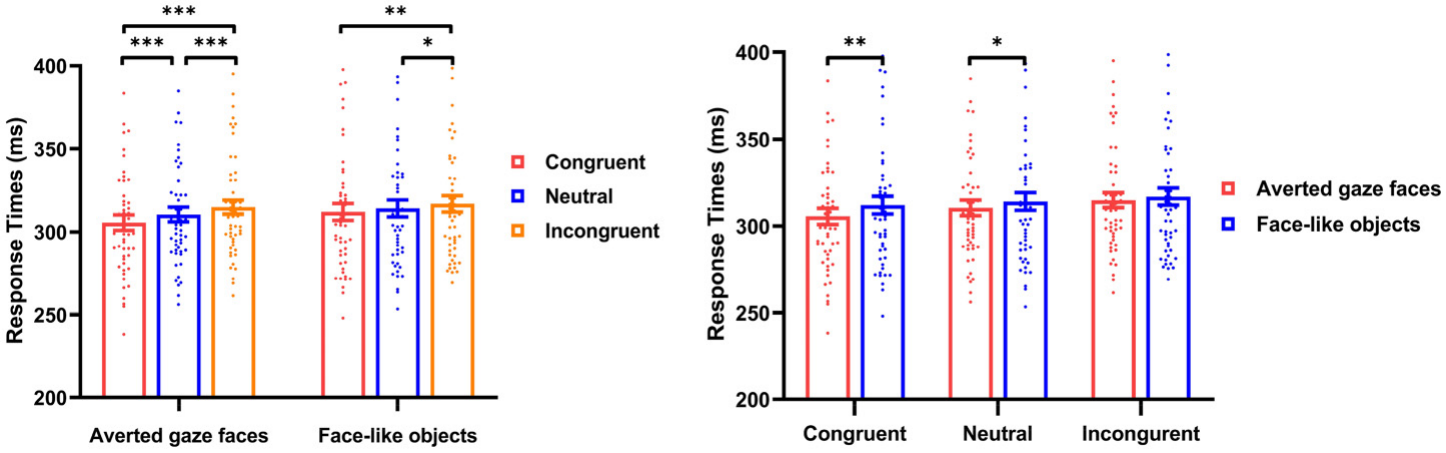
Results of mean RTs in Experiment 3. Mean RTs for the gaze cueing task as a function of stimuli type (averted gaze faces vs. face-like objects) and cue-probe congruency (congruent vs. neutral vs. incongruent). Error bars show standard errors. *** *p* < .001, ** *p* < .01, * *p* < .05.

*Note.* RT = response time.

For RTs, the ANOVA analysis revealed a significant main effect of stimuli type (averted gaze faces vs. face-like objects), *F*(1, 51) = 5.421, *p* = .024, $\eta_{p}^{2}$ = .096. Post hoc testing further elucidated that compared with face-like objects (314.31 ± 36.06 ms), participants responded significantly faster for averted gaze faces (310.25 ± 31.78 ms). And a significant main effect of cue-probe congruency (congruent vs. neutral vs. incongruent) was observed, *F*(2, 102) = 30.923, *p* < .001, $\eta_{p}^{2}$ = .377. Post hoc testing further elucidated that compared with incongruent trials (303.06 ± 32.54 ms), participants responded significantly faster both in congruent trials (290.01 ± 34.46 ms, *p* < .001) and in neutral trials (296.56 ± 33.77 ms, *p* < .001), and participants responded significantly faster in congruent trials than in neutral trials (*p* < .001). There was a significant interaction between stimuli type and cue-probe congruency, *F*(2, 102) = 6.625, *p* = .002, $\eta_{p}^{2}$ = .115. The mean RTs from six conditions are shown in Figure 4.

Considering the interaction between stimuli type and cue-probe congruency, a simple effect analysis revealed that for averted gaze faces, the RTs in congruent trials (305.55 ± 33.59 ms) were both lower than in neutral trials (310.40 ± 31.89 ms; *t*(51)  = –5.098, *p* < .001, *Cohen's d* = –0.707) and incongruent trials (314.80 ± 30.77 ms; *t*(51)  = –6.708, *p* < .001, *Cohen's d* = –0.930), and the RTs in neutral trials were lower than in incongruent trials (*t*(51) = –4.972, *p* < .001, *Cohen's d* = –0.689). For face-like objects, the RTs in congruent trials (312.00 ± 36.85 ms) were lower than in incongruent trials (316.86 ± 35.47 ms; *t*(51) = –3.626, *p* = .002, *Cohen's d* = –0.503), and the RTs in neutral trials (314.08 ± 36.65 ms) were lower than in incongruent trials (*t*(51) = –2.867, *p* = .018, *Cohen's d* = –0.398). Furthermore, we also found the RTs for averted gaze faces were lower than for face-like objects both in congruent trials (*t*(51) = –3.124, *p* = .003, *Cohen's d* = –0.433) and in neutral trials (*t*(51) = –2.107, *p* = .040, *Cohen's d* = –0.292).

**Figure 5.**
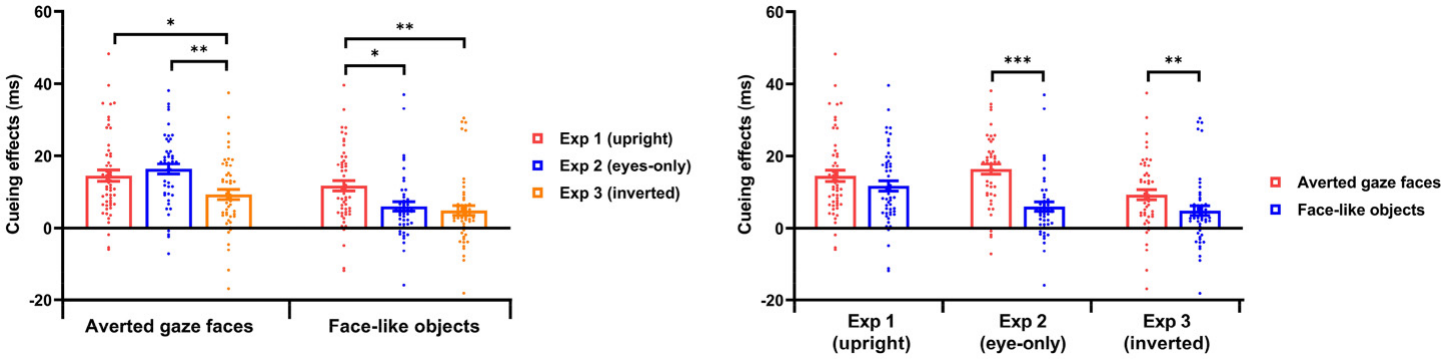
Result of cueing effects in cross-experiment comparison. Cueing effects for the gaze cueing task as a function of stimuli presenting mode (upright vs. eye-only vs. inverted) and stimuli type (averted gaze faces vs. face-like objects). *** *p* < .001, ** *p* < .01, * *p* < .05. Error bars show standard errors.

For cueing effects, the ANOVA analysis revealed a significant main effect of stimuli type (averted gaze faces vs. face-like objects), *F*(1, 151) = 42.317, *p* < .001, $\eta_{p}^{2}$ = .219. And a significant main effect of stimuli presenting mode (upright vs. eye-only vs. inverted) was observed, *F*(2, 151) = 6.971, *p* = .001, $\eta_{p}^{2}$ = .085. There was a significant interaction between stimuli type and stimuli presenting mode, *F*(2, 151) = 6.412, *p* = .002, $\eta_{p}^{2}$ = .078. The mean RTs from six conditions are shown in Figure 5.

Considering the interaction between stimuli type and stimuli presenting mode, a simple effect analysis revealed that for averted gaze faces, the cueing effects on the upright mode (14.43 ± 11.35 ms) were greater than on the inverted mode (9.25 ± 9.95 ms; *t*(104) = 2.494, *p* = .035, *Cohen's d* = .485), and the cueing effects on the eye-only mode (16.30 ± 9.80 ms) were greater than on the inverted mode (*t*(98) = 3.564, *p* = .003, *Cohen's d* = .713). For face-like objects, the cueing effects on the upright mode (11.66 ± 10.39 ms) were greater than on the eye-only mode (5.96 ± 9.02 ms; *t*(100) = 2.942, *p* = .011, *Cohen's d* = .584) and the inverted mode (4.85 ± 9.65 ms; *t*(104) = 3.490, *p* = .001, *Cohen's d* = .678). Furthermore, we also found the cueing effects for averted gaze faces were higher than for face-like objects both on the eye-only mode (*t*(47) = 6.862, *p* < .001, *Cohen's d* = 0.990) and on the invited mode (*t*(51) = 3.242, *p* = .005, *Cohen's d* = 0.450).

### Discussion

In Experiment 3, cueing effects were observed for both types of inverted stimuli. Bartlett and Searcy (1993) proposed that inverting faces disrupted the coding of facial configuration. Therefore, participants in Experiment 3 processed the local features from inverted faces to make attentional shifts. It is further confirmed that the cueing effect of averted gaze faces mainly originates from the local process (Burra & Kerzel, 2021; Conty et al., 2006; Senju et al., 2005; Tanaka et al., 2022). However, unlike the attentional benefit observed with eye-like parts in Experiment 2, no such benefit was found in Experiment 3. This discrepancy may be due to the disruption of the face-like configuration in the inverted stimuli.

Using cross-experiment comparison, we found that the cueing effects of face-like objects were greater in Experiment 1 than in Experiments 2 and 3. Therefore, the difference in cueing effects produced by face-like objects across the three experiments might result from the lack of holistic processing. However, since participants in Experiments 1 to 3 were not the same, we cannot be certain whether the main effect of stimuli presentation mode observed across experiments was due to the cueing stimuli or participant differences. Therefore, we conducted Experiment 4.

## Experiment 4

Experiments 2 and 3 collectively demonstrate that individuals interpret orientation from face-like objects through both local and global processes. However, it remains debatable whether the global configuration facilitates local processing or provides directional information independently, based on visual inspection of the results from Experiments 1 to 3. Furthermore, the results of the cross-experiment comparison could not fully address this question. Therefore, Experiment 4 was conducted to examine the differences in cueing effects induced by these stimuli across three presentation modes.

### Material and Method

#### Participants

Forty-eight undergraduate and graduate students (25 self-identified as female, 23 as male, 0 as nonbinary; *M* ± *SD*_age_ = 23.23 ± 3.33 years; range 18–31) participated in Experiment 4. All participants were right-handed, of Asian ethnicity, reported having normal color vision, and provided informed consent.

#### Apparatus and Stimuli

**Figure 6.**
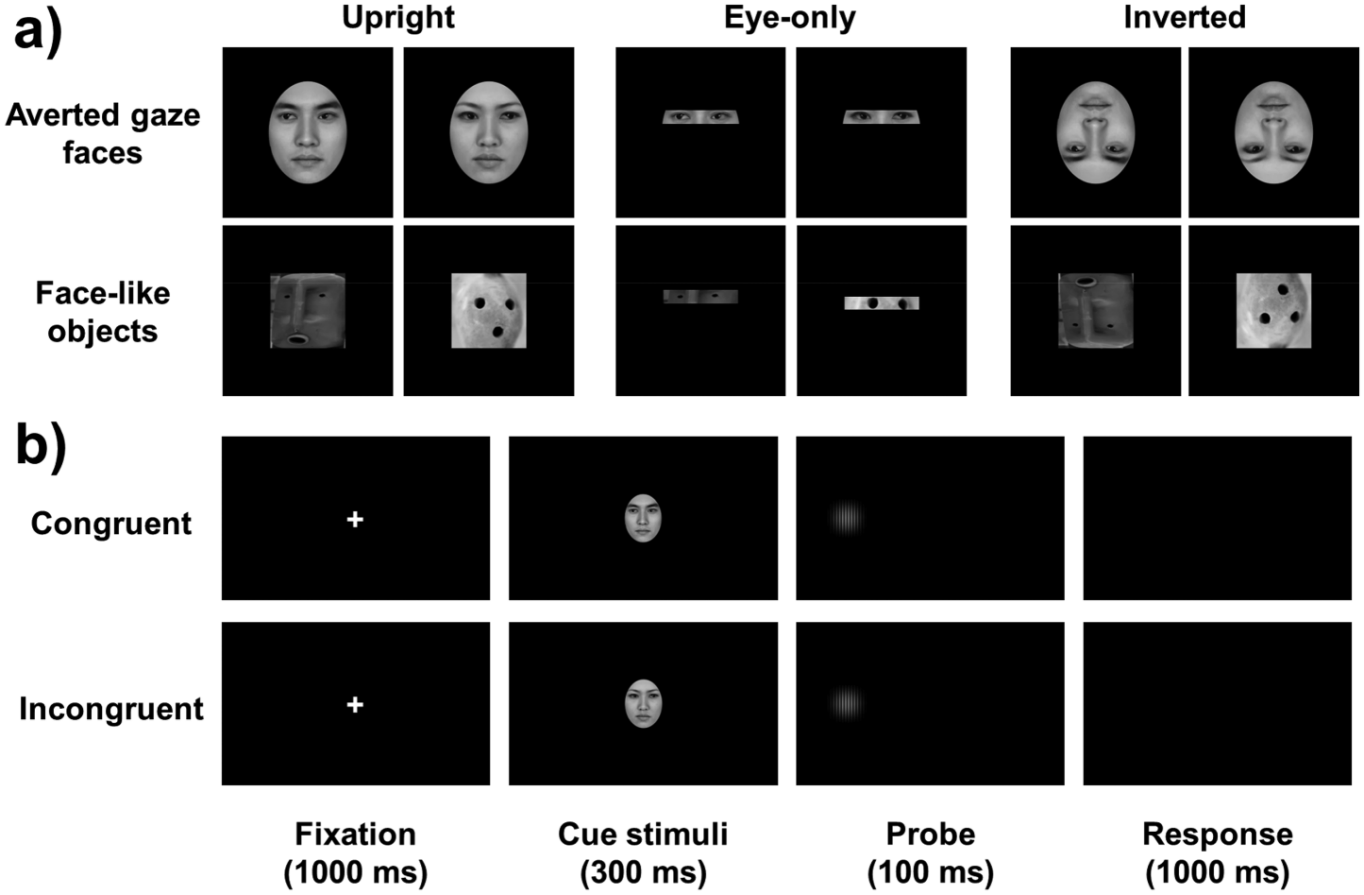
Flow diagram for Experiment 4. (a) The cueing stimuli used in these blocks, including averted gave faces and face-like objects. (b) The procedure for a single trial when using averted gaze faces as cueing stimuli.

Based on the results from experiments 1 to 3, we employed only two directions of cueing stimuli: averted gaze faces and face-like objects looking to the right or left side. Examples of the cueing stimuli are shown in Figure 6a.

#### Procedure

The procedure for one trial is shown in Figure 6b. There were three blocks, each containing one type of stimuli presenting mode (upright vs. eye-only vs. inverted). Each block consisted of 20 trials for practicing and 192 trials for testing. There were 96 congruent trials and 96 incongruent trials for testing in each block. The number of target-left and target-right trials was equal across congruent and incongruent trials. The number of averted gaze faces and face-like objects trials were equal in each block. The order of the three blocks was counterbalanced across participants. The entire experiment took about 20 min.

#### Data Analysis

The mean accuracy (ACC) was 96.95%. Therefore, we did not compare accuracy across conditions. For RT analysis, we excluded trials with RTs more than 3 SDs above or below the mean, resulting in an average exclusion of 1.63 trials per participant. Table 1 displays the mean RTs under each condition. Similar to the data analysis for cross-experiment comparison, cueing effects were subjected to a two-factor repeated-measures ANOVA with stimuli type (averted gaze faces vs. face-like objects) and stimuli presenting mode (upright vs. eye-only vs. inverted).

**Table 1.** Mean of average RTs (ms) in congruent trials and incongruent trials for each condition.

Condition		Averted gaze faces		Face-like objects	
Presenting mode	Cue-probe congruency	*M*	*SD*	*M*	*SD*
Upright	Congruent	324.15	34.74	324.15	34.74
	Incongruent	338.87	31.92	338.87	31.92
Eye-only	Congruent	321.67	34.73	321.67	34.73
	Incongruent	339.31	32.34	339.31	32.34
Inverted	Congruent	324.08	31.62	324.08	31.62
	Incongruent	333.55	32.82	333.55	32.82

RT = response time.

### Results

**Figure 7.**
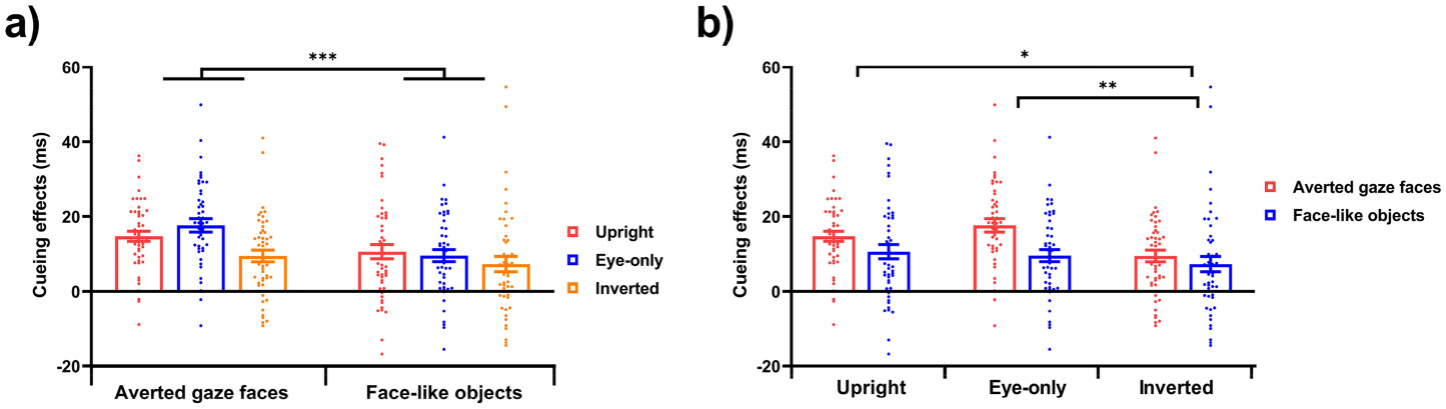
Result of cueing effects in Experiment 4. (a) Cueing effects for the gaze cueing task as a function of stimuli type (averted gaze faces vs. face-like objects). (b) Cueing effects for the gaze cueing task as a function of stimuli presenting mode (upright vs. eye-only vs. inverted). Error bars show standard errors. *** *p* < .001, ** *p* < .01, * *p* < .05.

For cueing effects, the ANOVA analysis revealed a significant main effect of stimuli type (averted gaze faces vs. face-like objects), *F*(1, 47) = 14.122, *p* < .001, $\eta_{p}^{2}$ = .231. Post hoc testing further elucidated that compared with face-like objects (9.15 ± 9.18 ms), cueing effects were higher for averted gaze faces (13.94 ± 7.46 ms). And a significant main effect of stimuli presenting mode (upright vs. eye-only vs. inverted) was observed, *F*(2, 94) = 6.089, *p* = .003, $\eta_{p}^{2}$ = .115. Post hoc testing further elucidated that compared with cueing effects on the inverted mode trials (8.37 ± 10.34 ms), cueing effects were higher on the upright mode (12.66 ± 9.35 ms, *p* = .026) and eye-only mode (13.60 ± 8.92 ms, *p* = .005). There was no significant interaction between stimuli type and stimuli presenting mode, *F*(2, 94) = 2.157, *p* = .121, $\eta_{p}^{2}$ = .044. The cueing effects from six conditions are shown in Figure 7.

### Discussion

The results revealed that cueing effects for averted gaze faces were greater than for face-like objects. Additionally, cueing effects on the inverted mode were lower than on the upright mode and the eye-only mode. We further discussed these results in the general discussion.

## General Discussion

We conducted four experiments to explore the similarities and differences in attentional shifts induced by averted gaze faces and face-like objects. In Experiment 1, cueing effects (RTs in congruent trials < RTs in incongruent trials) were observed for both types of stimuli. Subsequently, in Experiment 2, we focused solely on the eye regions of the stimuli, and in Experiment 3, we inverted the stimuli to disrupt their facial configuration. Cueing effects persisted across both types of stimuli in all three experiments. To examine differences in these cueing effects, we conducted Experiment 4. We found that cueing effects induced by averted gaze faces were greater than by face-like objects. Meanwhile, cueing effects on upright mode or eye-only mode were greater than on inverted mode.

Individuals interpret gaze direction from averted gazes to facilitate attentional shifts. In Experiments 1 to 3, cue-probe congruency resulted in both attentional benefit and cost for averted gaze stimuli. The results in Experiment 4 revealed that gaze cueing effects from upright averted gaze faces or from averted gazes were greater than those from inverted averted gaze faces, with no significant differences between upright averted gaze faces and averted gazes. Different from upright averted gaze faces, averted gazes lack facial configuration. These results suggest that the gaze cueing effect originates from local processing of the eye regions (Burra & Kerzel, 2021; Conty et al., 2006; Senju et al., 2005). Furthermore, different from allocating more attentional resources to process averted gaze, participants probably allocated a few resources to process the global configuration of inverted averted gaze faces. This misallocation of attentional resources might disrupt individuals’ gaze direction interpretation and weaken the gaze cueing effect (Jenkins & Langton, 2003; Schwaninger et al., 2005).

Similar to averted gazes, the cueing effects observed for upright face-like objects in Experiment 1 (attentional benefit and cost) and their eye-like parts in Experiment 2 (attentional benefit) suggest attentional orienting driven by local processing. The eye-like features of these objects closely resembled real eyes in shape and contrast (Caruana & Seymour, 2021; Epley et al., 2007; Kobayashi & Kohshima, 2001; Zhang et al., 2011), likely leading participants to associate them with averted gazes and incorporate associated social attributes (Bar, 2007; Chen et al., 2023; Epley et al., 2007; Hamza et al., 2022; Hull et al., 2023; Rekow et al., 2022). In contrast, inverted face-like objects produced an attentional cost (Experiment 1), potentially arising from processing the eye-like features themselves or from deriving orientation from low-level physical properties like luminance variations or asymmetry, akin to head orientation cues (Jenkins & Langton, 2003; Kobayashi & Kohshima, 2001; Schwaninger et al., 2005). Results from Experiment 4 were consistent with these interpretations. Our findings suggested that low-level physical properties did not affect the cueing effect triggered by face-like objects, whereas face-like configuration enhanced it. The object stimuli in upright mode contained eye-like features, face-like configuration and low-level physical properties. In contrast, the stimuli in eye-only mode contained only eye-like features, while those in inverted mode included both eye-like features and low-level physical properties. The results in Experiment 4 revealed that upright face-like objects elicited a stronger cueing effect than inverted face-like objects, likely due to the presence of face-like configuration. There were no significant differences in cueing effects between upright face-like objects and eye-like parts, suggesting that the absence of face-like configuration and basic physical attributes had minimal impact. Similarly, no significant differences were found between eye-like parts and inverted face-like objects, indicating that low-level physical properties did not affect the cueing effect. These findings highlight the crucial role of face-like configuration in enhancing the cueing effect (Chen et al., 2023; Epley et al., 2007; Rekow et al., 2022; Zhou et al., 2021). Impairment of the global process may hinder the local process required for attentional shifts. Overall, both eye-like parts and face-like configurations are processed to facilitate attentional shifts.

### Limitation

Nonetheless, it is crucial to acknowledge the limitations of this study. First, our participants were Asian college students, and we observed the cueing effect in response to averted-gaze faces and face-like objects within this specific demographic. A recent study found that subtle cultural differences (Italian vs. German) could shape face pareidolia (Romagnano et al., 2023). Easterners, for example, tend to prioritize the global configuration of faces more than Westerners (McKone et al., 2010). Given that the attentional shifts triggered by averted gaze faces and face-like objects stem from local processing, it is plausible that there may be cross-cultural variations in attentional shifts induced by these two stimuli. Second, according to previous research (Ji et al., 2020; McCrackin & Itier, 2018; Qian et al., 2013), the pictures of averted gaze faces were cropped into an oval shape to prevent the impact of neck and hair. Considering that the oval shape was similar to the facial configuration, we presented pictures of face-like objects cropped into a square rather than an oval shape. This difference in shape might have caused the face-like objects to contain less salient social information than the averted gaze faces (Hadjikhani & Johnels, 2023). Compared to the study by Takahashi and Watanabe (2013), our study balanced the orientation of the two stimuli in the preliminary experiment (see Supplemental Information), but did not balance their luminance for maintaining the ecological validity. Furthermore, given that direct gaze captures attention (Senju & Hasegawa, 2005), it would be preferable to avoid using cueing stimuli in neutral trials.

Critically, a fundamental limitation of our design lies in the simultaneous variation of stimulus type (real faces vs. face-like objects) and directional cue type (gaze direction vs. object orientation). This confound fundamentally constrains the interpretability of our findings, as it prevents us from determining whether observed differences in cueing effects are attributable to the social nature of the stimuli themselves or to the specific directional information from object orientation. Balancing the tradeoff between removing eye-like parts and preserving the face-like configuration presents a further challenge in isolating whether the face-like configuration itself produces the cueing effect or merely facilitates the effect produced by eye-like parts. Future studies should therefore adopt a more effective approach, simultaneously comparing the cueing effects of face-like objects, objects devoid of both face configuration and features, and averted gaze faces. Importantly, such comparisons should also consider incorporating nonsocial directional cues (e.g. arrows), particularly given evidence that their cueing effects may themselves stem from internalized social learning acquired early in development (Chacón-Candia et al., 2023; Gregory & Jackson, 2017; Hommel et al., 2001). Examining the relative influence of these diverse cues on attentional shifts will be crucial for disentangling domain-general attentional mechanisms from those specifically tuned to social information.

## Conclusions

In conclusion, our findings demonstrated that individuals could make attentional shifts by processing the local features of averted gaze faces and face-like objects. Moreover, we proposed that the face-like configuration of objects enhances attentional shifts triggered by eye-like features. This study suggested potential avenues for future research on the role of global and local processes in attentional shifts triggered by face-like stimuli. This study can provide a theoretical basis for understanding the processing mechanisms of face pareidolia, shedding light on why individuals make attentional shifts when seeing face-like objects (Atkinson et al., 2018). The findings also have practical implications for utilizing face pareidolia in product advertising (Delbaere et al., 2011; Guido et al., 2018; Noble et al., 2023). For example, advertizers can incorporate face-like configurations to enhance the salience of eye-like parts, thereby increasing consumers’ attentional shifts or captures toward their advertisements and leaving a lasting impression of their products.

## Supplemental Material

sj-docx-1-ipe-10.1177_20416695251352129 - Supplemental material for How face-like objects and averted gaze faces orient our attention: The role of global configuration and local features Supplemental material, sj-docx-1-ipe-10.1177_20416695251352129 for How face-like objects and averted gaze faces orient our attention: The role of global configuration and local features by Ziwei Chen, Mengxin Wen, Xun Liu and Di Fu in i-Perception
