# Supplementary material for: How face-like objects and averted gaze faces orient our attention: The role of global configuration and local features
Source: Iperception. 2025 Jul 23;16(4):20416695251352129. doi: 10.1177/20416695251352129 (PMC12350056; doi:10.1177/20416695251352129)
Supplement: sj-docx-1-ipe-10.1177_20416695251352129 - Supplemental material for How face-like objects and averted gaze faces orient our attention: The role of global configuration and local features [file sj-docx-1-ipe-10.1177_20416695251352129.docx]

**Supplementary Information**

1. Preliminary Experiment

Prior research found that a more pronounced gaze direction results in a stronger gaze-cueing effect (Yokoyama & Takeda, 2019; Zhang et al., 2011). Consequently, researchers would control the perceived gaze direction when using averted gaze faces as cueing stimuli (Bayliss et al., 2011; Qian et al., 2013). Similarly, Palmer and Clifford (2020) controlled the gaze direction of face-like objects. They found that these face-like objects could produce sensory-adaptation effects similar to those of averted gaze faces. However, when testing the attentional shifts triggered by face-like objects, Takahashi and Watanabe (2013) found no differences in cueing effect between face-like objects and schematic faces. A critical factor that may explain the absence of observed differences is the lack of gaze direction control for face-like objects. Therefore, we recruited volunteers to assess the gaze direction of averted gaze faces and face-like objects, ensuring precise control over the gaze direction of these stimuli for use in subsequent experiments.

1. Material and methods
2. Participants

Twenty-five undergraduate and graduate students (17 self-identified as female, 8 as male, 0 as non-binary; *M* ± *SD* _age_ = 23.84 ± 2.49 years; range 19-29) participated in this study. All participants were right-handed, of Asian ethnicity, reported normal color vision, and provided informed consent.

1. Apparatus and Stimuli

This experiment was performed using E-prime 2.0 (Psychology Software Tools, Inc., Sharpsburg, PA, USA) for programming and data recording. Stimuli were presented on a 21.5-inch Dell display (1024 × 768 at 60 Hz), 32-bit color depth.

For averted gaze faces, we collected 40 neutral Asian composite faces (half male and female) from the website (<https://generated.photos>). Then, these faces were rendered into 3D photography of human models using Facegen Modeller 3.5 (<http://facegen.com>). All face stimuli were frontally oriented toward the viewer without hair. The face stimuli, created from each character, were positioned in one of nine directions by setting the eye gaze modifiers from the Morph bar, denoted as 'modifier-lookleft' and 'modifier-lookright', with values ranging from -1 to 1 (see Figure S1a). Negative values indicated a leftward shift of the eyes from the viewer's perspective, while positive values signified a rightward shift. Other modifiers, such as modifier-lookup and modifier-lookdown, were set to zero. In male models, the mean value for 'Eyes-down/up' was 0.54 ± 0.70, 'Eyes-small/large' was -0.31 ± 0.37, 'Eyes-tilt inward/outward' was 0.23 ± 0.45, and 'Eyes-apart/together' was -0.73 ± 0.55. In female models, the mean value for 'Eyes-down/up' was 0.98 ± 0.74, 'Eyes-small/large' was -0.07 ± 0.50, 'Eyes-tilt inward/outward' was 0.27 ± 0.69, and 'Eyes-apart/together' was -0.88 ± 0.60. Each face stimulus was rendered against a uniform black background. A single model yielded nine pictures, with each pair of eyes looking in one of nine different directions, resulting in a total of 360 pictures of averted gaze faces. These pictures were standardized in terms of brightness and contrast. For face-like objects, we collected these from the public domain using Google Images (<https://www.flickr.com>) with the search terms "face-like objects" or "face pareidolia" (such as house, socket and bread; see Figure S1b). Ninety-nine pictures of face-like objects were collected, including 42 pictures suspected of looking at the left or right side, and 15 pictures suspected of looking directly. The types of face-like objects contained both natural and artificial objects. A total of 459 materials were used for the rating, all 625 × 625 pixel JPG files with 8-bit color depth.


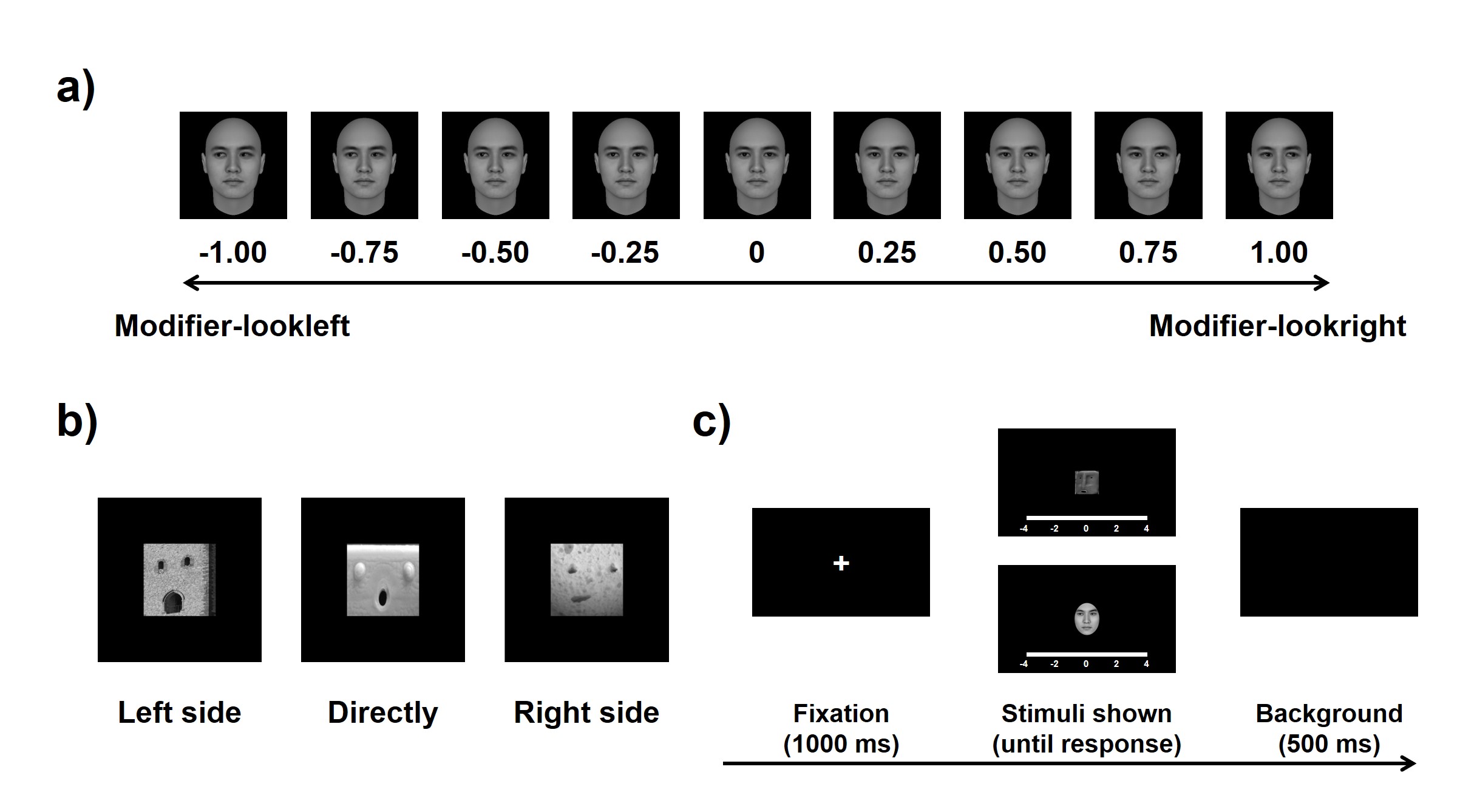


*Figure S1.* In preliminary experiment, the gaze directions of averted gaze faces and face-like objects were rated. a) Sample graphs of the averted gaze faces in nine different directions generated by one model. b) Examples of face-like objects looking in three different directions. c) The procedure for a single trial in this experiment.

1. Procedure

In this experiment, participants were seated approximately 50 cm away from the screen and required to perceive the gaze directions of the averted gaze faces and face-like objects. The procedure of this experiment is shown in Figure S1c. Each trial started with a fixation "+" (1.1° × 1.1°), lasting for 1000 ms in the center of the screen. Then, a cue stimulus (9.1° × 9.1°) was displayed in the center of the screen. Meanwhile, a rating bar (29.8° horizontal × 0.9° vertical, the score was from -200 to 200) was displayed at the bottom of the screen. Participants were required to report the gaze directions of the averted gaze faces and face-like objects by moving the mouse to select a point on the rating bar. The corresponding numbers (-4, -2, 0, 2, 4) were marked equidistantly on the rating bar for reference. After they rated one stimulus, the screen was blank for 500 ms before the subsequent trial. Preliminary experiment consisted of a practice phase with eight trials and a formal phase with 459 trials. The pictures used in the practice phase were additional eight averted gaze faces. In the formal phase, a total of 459 pictures were presented randomly and only once.

1. Results

For averted gaze faces generated by FaceGen Modeller 3.5, the mean distances of averted gaze faces are shown in Figure S2. A linear function was fitted to these data to capture the relationship between the eye gaze modifiers and the mean distances. The resulting best-fit line had a slope of 1.11 and an intercept of −14.34. There was a significant positive correlation between the eye gaze modifiers and the mean distances, *r*(360) = .96, *p* < .001, indicating that averted gaze faces with larger eye gaze modifiers were rated as higher distances.


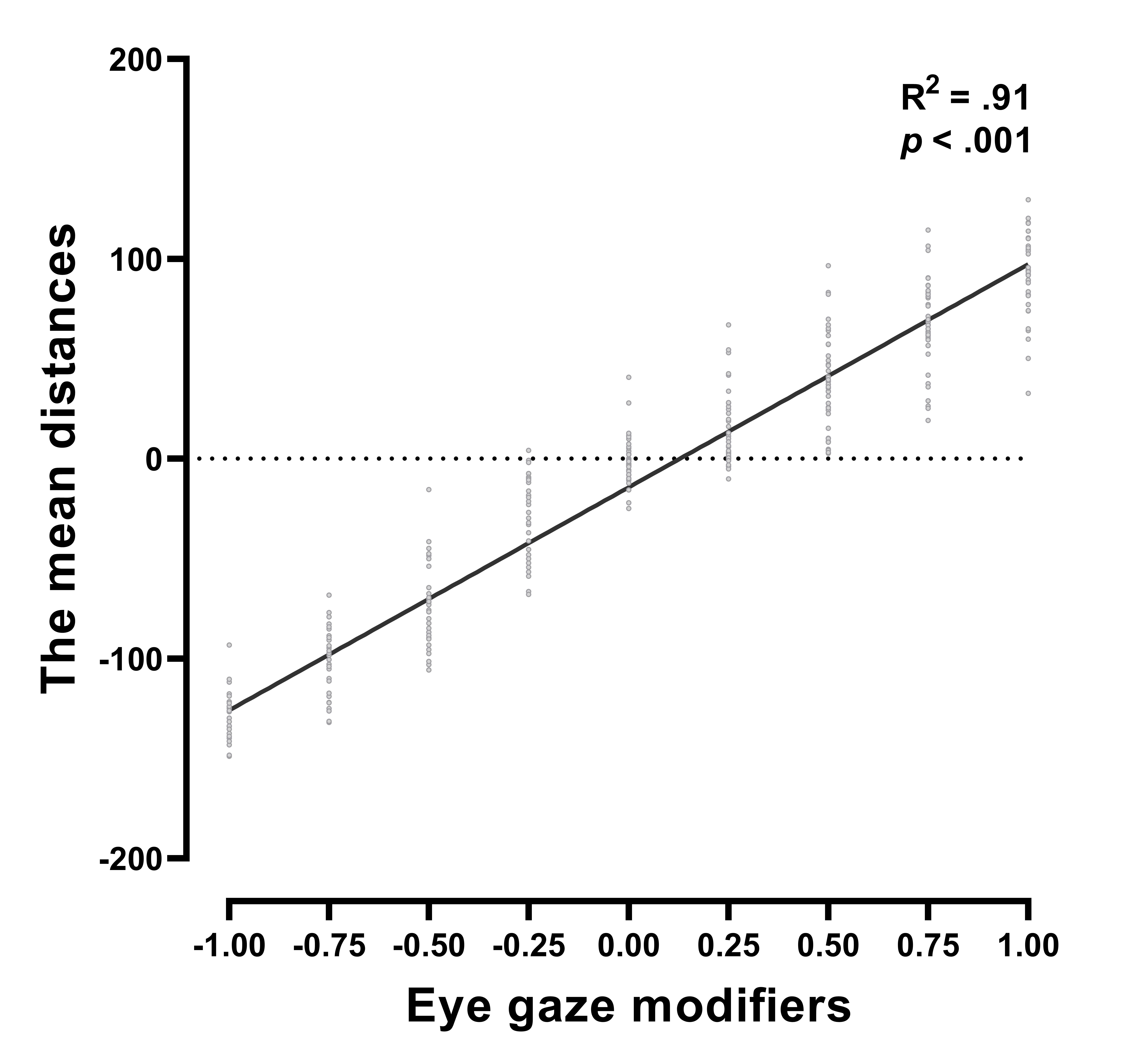


*Figure S2.* The relationship between the objective index (eye gaze modifier) and the subjective index (distance) of averted gaze faces.

Using this psychophysical task, we used a portion of materials as three types of averted gaze face and face-like object stimuli: looking directly to the viewer and correspondingly at the right or left side (see Figures S3a & S3b). In averted gaze face stimuli, each type consisted of 12 faces (half male faces and half female faces). In face-like object stimuli, each type consisted of 12 objects. The mean distances of stimuli were subjected to a two-factor ANOVA with stimuli type (averted gaze faces vs. face-like objects) and gaze direction (looking at the left side vs. looking directly vs. looking at the right side). The ANOVA analysis revealed an insignificant main effect of stimuli type, *F*(1, 66) = 0.046, *p* = .830, *η_p_*^²^ = .001. However, a significant main effect of gaze direction was observed, *F*(2, 66) = 1243.132, *p* < .001, *η_p_*^²^ = .974. The post hoc test indicated that there was a significant difference among the three conditions of gaze direction (*M* _left side_ = -80.03 ± 2.75, *M* _directly_= -1.79 ± 1.29, *M* _right side_ = 85.94 ± 2.63; all *p*s < .001). There was no significant interaction between stimuli type and gaze direction, *F*(2, 66) = 0.419, *p* = .660, *η_p_*^²^ = .013.





*Figure S3.* The mean distance of averted gaze faces and face-like objects as cueing stimuli for subsequent experiments. a) The bar graph for the mean distance of averted gaze face stimuli. b) The bar graph for the mean distance of face-like object stimuli. Error bars show standard errors.

References

Bayliss, A. P., Bartlett, J., Naughtin, C. K., & Kritikos, A. (2011). A direct link between gaze perception and social attention. *Journal of Experimental Psychology-Human Perception and Performance*, *37*(3), 634-644. <https://doi.org/10.1037/a0020559>

Palmer, C. J., & Clifford, W. G. C. (2020). Face pareidolia recruits mechanisms for detecting human social attention. *Psychological Science*, *31*(8), 1001-1012. <https://doi.org/10.1177/0956797620924814>

Qian, Q., Song, M., & Shinomori, K. (2013). Gaze cueing as a function of perceived gaze direction. *Japanese Psychological Research*, *55*(3), 264-272. <https://doi.org/10.1111/jpr.12001>

Takahashi, K., & Watanabe, K. (2013). Gaze cueing by pareidolia faces. *i-Perception*, *4*(8), 490-492. <https://doi.org/10.1068/i0617sas>

Yokoyama, T., & Takeda, Y. (2019). Gaze cuing effects in peripheral vision. *Frontiers in Psychology*, *10*, 708, Article 708. <https://doi.org/10.3389/fpsyg.2019.00708>

Zhang, Z., Zhao, Y., & Zhan, Q. (2011). Effects of gaze direction perception on gaze following behavior. *Acta Psychologica Sinica*, *43*(7), 726-738, Article 0439-755x(2011)43:7<726:zsfxdz>2.0.tx;2-a. <Go to ISI>://CSCD:4235827
